# Increased absorptive transcytosis and tight junction weakness in heart failure are equally corrected by exercise training and losartan

**DOI:** 10.1042/CS20242965

**Published:** 2025-05-29

**Authors:** Hiviny de Ataides Raquel, Mariana Makuch-Martins, Sany M. Perego, Gustavo S. Masson, Leonardo Jensen, Lisete C. Michelini

**Affiliations:** 1 Department of Physiology and Biophysics, Biomedical Sciences Institute, University of Sao Paulo, São Paulo, Av. Prof. Lineu Prestes, 1524, SP 05508-000Brazil; 2 School of Medicine, University of Sao Paulo, Sao Paulo, Brazil

**Keywords:** heart failure, angiotensin II, blood–brain barrier, tight junctions, absorptive transcytosis, microglia

## Abstract

Reduced ventricular function, renin–angiotensin system up-regulation, and sympathoexcitation are hallmarks of heart failure (HF). Recently, we showed that blood–brain barrier (BBB) lesion within autonomic nuclei contributes to autonomic imbalance and that exercise training (T) normalizes BBB function and improves autonomic control. We sought now to identify the mechanism(s) involved in both HF-induced lesion and exercise-induced correction. Wistar rats submitted to coronary artery ligation were, after the development of HF, assigned to losartan (Los) or vehicle (Veh) treatments and simultaneously submitted to T or sedentary (S) protocol. After hemodynamic/autonomic recordings and evaluation of BBB permeability, brains were harvested for ultrastructural analyses of the barrier (tight junctions’ [TJs] tightness and vesicles trafficking) within capillaries of the hypothalamic paraventricular nucleus. Local angiotensin II (Ang II) expression and activation of microglial cells (IBA-1 immunofluorescence) were also evaluated. High sympathetic activity and pressure variability, reduced parasympathetic control of the heart, elevated BBB permeability, high vesicular trafficking, and TJ weakness exhibited by Veh-S rats were equally corrected in Veh-T, Los-S, and Los-T groups. The increased paraventricular hypothalamic nucleus (PVN) Ang II expression and IBA-1 density in Veh-S group were similarly reduced by T, Los and combination of both. Ang II, colocalized with microglia AT1 receptors, induced their remodeling from disease-associated phenotype in Veh-S rats to homeostatic-surveilling conditions in the other groups. All measured parameters exhibited strong correlations with Ang II availability. Data indicated that changes in PVN Ang II availability induced by HF, exercise, and Los are the key regulator of transcellular and paracellular transport across the BBB.

## Introduction

Heart Failure (HF) is a life-threatening condition characterized by the inability of the heart to adequately supply blood for metabolic demands of the body [1]. Cardiac dysfunction is accompanied by hemodynamic abnormalities, up-regulation of the renin–angiotensin system (RAS), impaired baroreflex sensitivity, autonomic imbalance, and sympathoexcitation, with high prevalence of morbidity/mortality [1–5]. It was also shown that HF is a low-grade inflammatory disease leading to microglia activation and the release of pro-inflammatory cytokines within autonomic, emotional, and cognitive nuclei, which contribute to cardiovascular dysregulation, anxiety, depression, and cognitive disorders [6–8]. In a recent study on blood–brain barrier (BBB) function in HF rats, we documented a significant reduction in capillaries’ lumen diameter accompanied by increased basement membrane thickness and a dysfunctional barrier with high permeability within three important autonomic nuclei (paraventricular hypothalamic nucleus [PVN], nucleus of the solitary tract, and rostral ventrolateral medulla) [9]. BBB dysfunction, caused by augmented vesicles’ trafficking (transcytosis) across the endothelial cell and reduced tight junctions’ (TJs) tightness, was positively correlated with vasomotor sympathetic activity and pressure variability [9].

Regular exercise training was shown to be associated with health-improving effects in HF patients as well as in animal models [10,11]. Analyzing the effects of exercise training within autonomic areas of HF rats, we also observed that exercise increases basement membrane thickness, reduces capillaries’ lumen diameter, and normalizes the transport mechanisms across the BBB, thus correcting barrier permeability and abrogating the sympathoexcitation [9]. In spontaneously hypertensive rats (SHR), we recently documented that the initial stimulus to trigger BBB lesion within autonomic nuclei was the increased local expression of angiotensin II (Ang II) that disrupts the barrier, which allows the entrance of plasma Ang II with great increment of the peptide within the brain parenchyma [12]. The dependence of BBB disruption on brain Ang II availability was already demonstrated in chronic hypertensive rats [13,14]. It was also shown that exercise-induced down-regulation of vasoconstrictor axis of brain RAS is the main effect to restore autonomic dysfunction in SHR and that the correction of BBB lesion is abrogated in trained SHR simultaneously infused with intracerebroventricular Ang II [14,15]. Based on these evidences, we hypothesized that Ang II availability within autonomic nuclei is an important regulator of BBB disruption in HF (high levels) as well as its correction by exercise training (low levels). Therefore, we analyzed within the PVN (an important integrative autonomic nucleus) of infarcted rats the effects of exercise training on local Ang II expression, hemodynamic and autonomic parameters, BBB permeability, and ultrastructure of brain capillaries with special attention to transcytosis and TJs’ ultrastructure. Considering that pro-inflammatory cytokines synthetized by activated microglial cells contributed to sympathoexcitation in HF [6,8,11] and that there was no information on the effects of exercise on microglia activity, additionally, we evaluated in trained rats the structural remodeling of microglial cells. As a proof of concept, the above-cited effects were also analyzed in sedentary and trained HF rats simultaneously treated with losartan (Los) or vehicle (Veh).

## Methods

### Animals care and induction of myocardial infarction

Male Wistar rats (eight weeks old, 250–300g) were housed in collective cages (four/cage) on a 12–12h light-dark cycle under controlled temperature (22–24°C) in the Animal Facilities of the Department of Physiology and Biophysics, Biomedical Sciences Institute, University of Sao Paulo. Standard chow and tap water were provided *ad libitum*.

Rats were sedated with the pre-anesthetic agent acepromazine *sc*. (2.5 mg/kg) and 30 min later anesthetized with ketamine hydrochloride (100 mg/kg, Cetamin 10%, Syntec®, Cotia, Brazil) plus xylazine hydrochloride *ip*. (10 mg/kg, Xilasin 2%, Syntec®). They are then submitted to orotracheal intubation plus artificial ventilation (Harvard Apparatus®, model 683, South Natick, MA, U.S.A., 2.5 ml/insufflation). The induction of HF was performed as previously described [9,16]. Briefly, after the left thoracotomy and exposition of the heart, the anterior descending coronary artery was isolated and ligated. The chest was closed and the pneumothorax drained. Except for coronary obstruction, the same procedure was performed in a small group of Sham rats just to compare and evaluate the development of HF in rats submitted to coronary artery ligation. Rats were treated subcutaneously twice-a-day with ketoprofen (Biofen, Biofarm®, 5 mg/kg, Jaboticabal, Brazil) and enrofloxacin (Baytril, Bayer®, 10 mg/kg, São Paulo, Brazil), placed in individual cages under heating (24°C) and monitored until full recovery from anesthesia. The antibiotic and analgesic treatments were extended for five days.

### Echocardiographic examination

Four weeks later, rats were anesthetized with ketamine + xylazine (80 mg/kg + 8 mg/kg, *i.p*.) and underwent to echocardiography to confirm the effectiveness of myocardial infarction. Left ventricle function was evaluated by a 4–12 MHz transducer (Vivid 9, GE Healthcare®, Fairfield, CT, USA). To guarantee the development of HF, only rats with an ejection fraction ≤42% were included in this study.

### Training and sedentary protocols

Two days after the echocardiographic examination, HF rats were adapted to walk/run on a treadmill (Millenium Inbramed® 0.4–0.7 km/h, 10–15 min/day, for five days). In order to determine individual exercise capacity and calculate the group training intensity, rats were then submitted to maximal exercise test (MET) in which mild exercise intensities were increased progressively [9,14–18]. HF rats with similar physical performance were allocated to training (T) or sedentary (S) protocol (week 0). T (50–60% of maximum intensity, 0% inclination) was performed 1 h/day, five days/week for eight weeks. The T protocol started with 50% intensity for short period of time and increased progressively attaining 1 h/day and 60% capacity by the end of third week. At the fourth week, when rats’ aerobic performance had increased, a second MET was performed to adjust exercise intensity to the new values of 50–60% capacity and other progressive increases in time and intensity were performed during four to eight weeks of the protocol. A third MET at the eighth week was used to assess the efficacy of T and S protocols. As confirmed by our previous studies, this protocol did not cause stress [9,14–18]. In order to expose to a similar environment, S rats were handled every day and performed a short walk on the treadmill (0.3 km/h, 10 min/day, 0% inclination) once a week. At the eighth experimental week, echocardiographic examination was again carried out in HF groups of rats.

### Los and Veh treatments

Concomitant with T and S protocols, HF rats were submitted to Los (= 20 mg/kg/day in drinking water) or Veh (= drinking water) treatment during eight weeks. Treatments (gavage) were carried out once a day, five days/week, approximately 2 h after the T session. Rats were weighed daily to adjust the dose and handled carefully by the researcher avoiding stressful/uncomfortable behaviors. Our experimental design consisted of four groups of HF rats: Veh-S, Veh-T, Los-S, and Los-T

### Assessment of cardiovascular parameters

After completing the experimental protocols, rats were anesthetized (acepromazine followed by ketamine + xylazine) for implantation of a catheter in the femoral artery, as previously described [17]. The catheter was exteriorized through the subcutaneous tissue in the back of the neck and fixed with suture. After surgery, rats were treated with ketoprofen and enrofloxacine and recovery in individual cages. On the following day, they were connected to the recording system (LabChart Pro 7, ADInstruments, Bella Vista, AU, sampling frequency of 2,000 Hz). Resting values of pulsatile arterial pressure (AP) and heart rate (HR) were recorded for 30–40 min in conscious freely moving rats on a beat-to-beat basis after the stabilization of cardiovascular parameters [17,18].

### Spectral analysis of cardiovascular parameters

Time series of systolic AP (SAP) and pulse interval (PI) were used to evaluate pressure and HR variabilities and the respective spectral components at the frequency domain using the Matlab® software (Matworks, Natrick, MA, U.S.A.), as previously described [18]. Power spectral density for low frequency (0.20–0.75 Hz, LF-SAP indicative of vasomotor sympathetic activity and LF-PI an index of sympathetic + parasympathetic activity to the heart) and high frequency (HF-PI>0.75–3.0 Hz, indicative of cardiac vagal modulation) were evaluated in the four experimental groups. The spontaneous baroreflex sensitivity (αHF) calculated by the square root of the ratio HF-PI (ms^2^)/HF-SAP (mmHg^2^, an index of spontaneous pressure fluctuations) was also evaluated.

### Analysis of BBB integrity

Analysis of BBB function was performed in anesthetized rats (ketamine + xylazine) after the hemodynamic recordings. The right carotid artery was dissected and a catheter was inserted in the cranial direction [14]. A mixture of fluorescent dyes (rhodamine isothiocyanate dextran, RHO-70kDa and fluorescein isothiocyanate dextran, FITC-10kDa, Sigma Aldrich) was slowly infused intra-arterially [13]. Dyes were allowed to recirculate for 20 min and rats were killed by an overdose of anesthesia for brain harvesting immediately after the respiratory arrest. Brains were collected, post-fixed (4% paraformaldehyde for 24 h), and cryoprotected (30% sucrose in 0.1 M PBS for three days at 4°C). Sequential coronal sections (30 μm, Leica®, CM 3050 cryostat, Germany) of the entire PVN length [19] were collected and analyzed as previously described [9,20]. Briefly, acquired images (Image-Pro Plus, Media Cybernetics) were examined by a blind observer on a fluorescence microscope (Leica® DMLB, Germany) attached to an ExiBlue camera (Imaging, Canada) and analyzed by the ImageJ according to the technique described by Biancardi et al. [13]. BBB permeability, evaluated by the capability of small size FITC-10kDa to remain inside the intact capillaries or partially leak into the brain parenchyma in the presence of compromised barrier integrity, was quantified bilaterally (five to eight slices/rat, three rats/group) in a specific ROI superimposed over the ventromedial nucleus of the PVN (PVN*vm*).

### Ultrastructural analysis of BBB constituents

After the functional measurements, Veh-S, Veh-T, Los-S, and Los-T rats received an overdose of ketamine + xylazine. Immediately after the respiratory arrest, the thorax was opened and the left ventricle cannulated for perfusion with Karnovsky solution and brain harvesting. The PVN tissue was carefully microdissected and processed for transmission electron microscopy as previously described [9,20]. Transverse sections of PVN*vm* capillaries (12–15 capillaries/rat, three rats/group) were acquired by the transmission electron microscope (Zeiss®, Leo 906E model, Germany) and analyzed using the ImageJ software. The following parameters were analyzed: lumen diameter, endothelial cell area, basement membrane thickness, pericytes' coverage of capillaries, occurrence of TJs, TJ/capillary border (quantified by the ratio between TJ extension to capillary border extension), and the counting of transcytotic vesicles/capillary. To avoid the inclusion of non-transcytotic vesicles, only vesicles being formed or attached to luminal and abluminal membranes were counted and expressed as number/capillary [9,20].

### Immunofluorescence assays

Other groups of Veh-S, Veh-T, Los-S, and Los-T rats were sacrificed by overdose of anesthetics and used for immunofluorescence assays. Brains were perfused and processed as previously described [9,21]. Briefly, after perfusion with Dubecco’s Modified Eagle’s Medium followed by the fixative, brains were removed, post-fixed, and cryoprotected. Coronal sections of the PVN (30 µm, Leica®, CM 3050 cryostat, Germany) were collected and stored in antifreeze solution until processing. For the immunofluorescence assays, free-floating sections pretreated with 1% H_2_O_2_, washed with 0.02M KPBS buffer, and incubated with 5% normal donkey serum were used [9]. Sections suspended in 0.3% Triton X-100 in 2% donkey serum were incubated for 24 h with a mixture of primary antibodies: polyclonal guinea pig anti-Ang II (1:250 dilution, BMA Biomedicals/Peninsula Laboratories, Cat. no. T-5001), an antibody generated by immunization with Ang II (100% cross-reaction with Ang II) that has been previously tested and validated by Elisa, showing very little cross-reaction with Ang I (0.8 %) and angiotensinogen (0.3 %) and polyclonal rabbit anti-ionized calcium-binding adapter molecule 1 (IBA-1, 1:1000 dilution, FujiFilm Wako Chemicals, Osaka Japan, Cat. no. 019–19741), a specific microglial marker raised against C-terminus of IBA-1 that do not cross-react with neurons and astrocytes [12]. Tissues were washed and submitted to 1 h incubation (0.02M KPBS, 0.3% Triton X-100, 1% donkey serum) at room temperature with secondary antibodies: anti-guinea pig Alexa Fluor 594 (Cat. No. 706–585-148) and anti-rabbit Alexa Fluor 488 (Cat. No. 711–545-152), 1:500 dilution each, Jackson ImmunoResearch, MD, U.S.A. Some slices suspended in 0.3% Triton X-100 in 2% donkey serum were also incubated for 48 h with polyclonal goat anti-Ang II type 1 receptor primary antibody (AT_1_R, 1:500 dilution, Santa Cruz Biotechnology, Cat. no. AT1 (N-10)-G: sc-1173-G), an antibody raised against a peptide mapping within the N-terminal extracellular domain of the human AT_1_R, followed by the incubation with the secondary antibody anti-goat Alexa Fluor 594, Cat. no. 705–585-003, 1:500 dilution, Jackson ImmunoResearch) for 90 min. Negative controls omitted the primary antibodies. Sections were washed and mounted in gelatinized slides plus cover slip with Prolong Gold Antifade (Thermo Fisher Scientific, Inc, Massachusetts, U.S.A.) and stored in dark at 4°C.

PVN sections were examined by a blind investigator in the fluorescence microscope (Leica® DMLB). Images were analyzed with identical acquisition settings; background fluorescence, threshold setting, and immunoreactivity quantification were made as previously described [21]. Both left and right sides, six slices/rat, four rats/group were quantified within the PVN*vm* with the same ROI used for BBB analysis; values were averaged to give a mean value for the nucleus for each rat in each condition. In the double-labelled slides, the association of Ang II with microglial cells was quantified using their binary images and the command ‘colocalization’ of the ImageJ. The colocalization of AT_1_R with microglia was also verified in one rat/group.

### Quantification of microglia morphology

**Figure 1:**
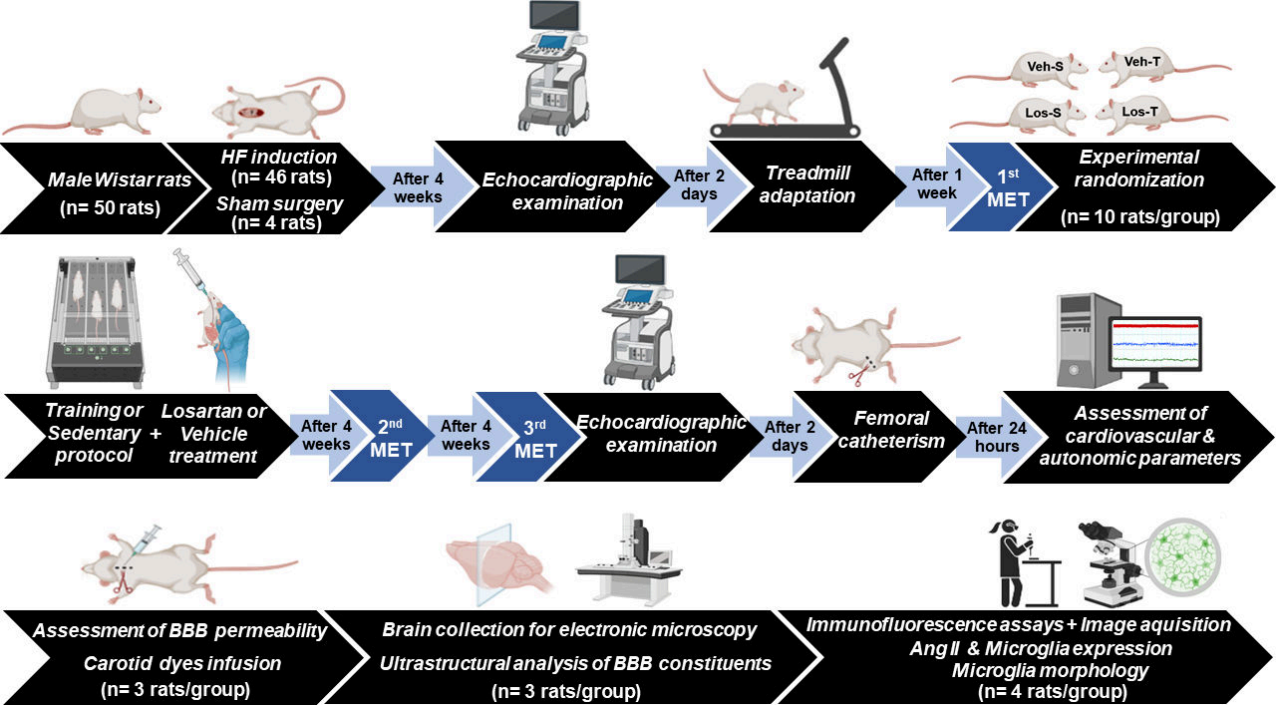
Timeline of experimental protocols. Some rats died after heart failure (HF) induction, others were excluded for not meeting the inclusion criteria and were killed by an overdose of ketamine + xylazine. MET, maximal exercise test performed on treadmill.

The effects of exercise and Los treatment on the morphologic phenotype of microglial cells within the PVN*vm* of HF rats were quantified by the NeurphologyJ [22], a freely available plug-in to ImageJ. The NeurphologyJ automatically analyzed the cell number, microglia processes number and length and their endpoints. In addition, the ratio between the total pixels within cell bodies and the respective number of microglial cells allowed us to calculate the individual soma size index [12]. A timeline of the entire experimental protocol is presented in Figure 1.

### Statistical analysis

Results are presented as means ± SEM. Data were subjected to the normality test (Kolmogorov Smirnov) and homogeneity of variances (Bartlett’s). Echocardiography parameters and treadmill performance were analyzed by three-way ANOVA for repeated measures (group, condition and time). Hemodynamic and autonomic parameters*,* BBB permeability, ultrastructural measurements of PVN capillaries, morphometric analysis of microglia, Ang II and IBA-1 protein expression, and their colocalization were analyzed by factorial two-way ANOVA with Tukey as the *post-hoc* test. Correlation analyses use Pearson statistics. All statistical analyses were performed using Prism® software (version 8), and the significance level adopted was *P*<0.05.

## Results

### Effects of myocardial infarction, Los treatment, and exercise training on ventricular function and hemodynamic/autonomic parameters

**Figure 2:**
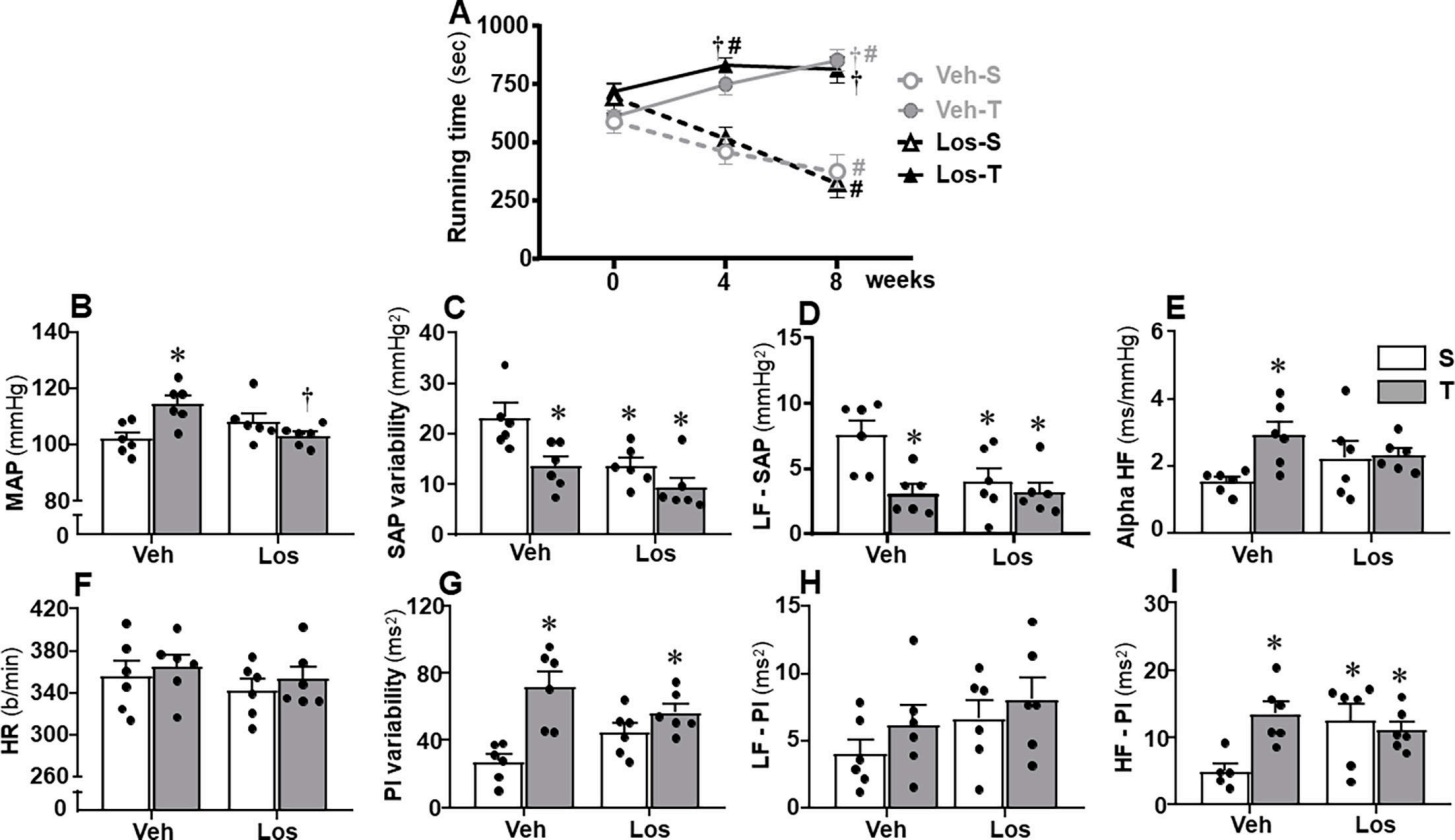
Treadmill performance and values of cardiovascular and autonomic parameters in heart failure rats treated with vehicle (Veh) or losartan (Los) and submitted to sedentary (**S**) or training (**T**) protocol. **(A)** Running time on treadmill: Comparisons made by three-way ANOVA with repeated measurements; *time* F(2,40)=4.27, *P*=0.027; *group* F(1,20)=0.96 , *P*=0.338; *condition* F(1,20)=37.69, *P*<0.001; *time × group* F(2,40)=6.26, *P*=0.004; *time* × condition F(2,40)=55.40, *P*<0.001; *group* × *condition* F(1,20)=0.02, *P*=0.884; *time × group × condition* F(2,40)=0.03, *P*=0.970. Significances (*P*<0.05): **^#^** *vs*. week 0;**^†^** *vs*. S. Bar graphs compare resting values of arterial pressure and heart rate, their variabilities and spectral components (*n* = 6 rats/group). Comparisons made by two-way factorial ANOVA. (**B)** MAP: *group* F(1,20) = 1.09, *P*=0.310; *condition* F(1,20) = 2.29, *P*=0.146; *interaction* F(1,20)=12.46, *P*=0.002. (**C)** SAP variability: *group* F(1,20) = 10.14, *P*=0.005; *condition* F(1,20) = 10.45, *P*=0.004; *interaction* F(1,20)=1.54, *P*=0.229. (**D)** LF-SAP: *group* F(1,20) = 3.90, *P*=0.062; *condition* F(1,20) = 9.02, *P*=0.007; *interaction* F(1,20)=4.28, *P*=0.051. (**E)** Alpha HF: *group* F(1,20) = 0.02, *P*=0.903; *condition* F(1,20) = 4.93, *P*=0.038; *interaction* F(1,20)=3.82, *P*=0.065. (**F)** HR: *group* F(1,20) = 1.25, *P*=0.277; *condition* F(1,20) = 3.31, *P*=0.587; *interaction* F(1,20)=0.06, *P*=0.814. (**G)** PI variability: *group* F(1,20) = 0.02, *P*=0.887; *condition* F(1,20) = 20.26, *P*<0.001; *interaction* F(1,20)=6.86, *P*=0.016. (**H)** LF-PI: *group* F(1,20) = 2.56, *P*=0.125; *condition* F(1,20) = 1.57, *P*=0.225; *interaction* F(1,20)=0.06, *P*=0.816. (**I)** HF-PI: *group* F(1,20) = 4.56, *P*=0.045; *condition* F(1,20) = 2.47, *P*=0.132; *interaction* F(1,20)=5.69, *P*=0.027. Significances (*P*<0.05):^*^vs. Veh-S; **^†^** *vs*. Veh-T

Infarcted rats had developed HF 4 after ligation of the anterior descending coronary artery, before starting the Los or Veh treatment and the trained or sedentary protocol (week 0). As depicted in Table 1, reduced left ventricle ejection fraction (LVEF) and fractional shortening (FS) and augmented systolic/diastolic diameters (LVSD/LVDD) observed in all experimental groups certified the development of HF. Indeed, LVEF and FS values were on average 52% and 60% lower, and LVSD and LVDD were 86% and 26% higher than values of age- and weight-matched rats submitted to sham surgery at week 0 (LVEF = 80.71±2.62%; FS = 45.16 ± 2.58%; LVSD = 4.14 ± 0.36 mm; LVDD = 7.53 ± 0.56 mm, *n* = 4). Sedentary HF rats treated with Veh exhibited a mild LVDD increase after eight weeks, an effect not observed in the other experimental groups whose echocardiographic parameters were similar to those observed at week 0. Sedentary HF rats treated with Veh or Los also showed after eight weeks, a large decrease in exercise capacity, whereas exercise training not only blocked this effect but also caused in both groups a small significant increase in the performance gain (Figure 2A)

**Table 1:** Characterization of heart failure by echocardiography in rats with anterior descending artery occlusion treated with vehicle (Veh) or losartan (Los) and submitted to aerobic training (T) or sedentary protocol (S) for eight weeks.

Echocardiographic parameters	Veh-S	Veh-T	Los-S	Los-T
**LVEF (%**) week 0 week 8	38.71 ± 0.57 41.14 ± 4.21	39.14 ± 1.01 40.29 ± 1.30	34.57 ± 2.96 41.29 ± 1.30	41.67 ± 1.28 49.33 ± 4.13
**LVSD (mm**) week 0 week 8	7.00 ± 0.27 7.86 ± 0.46	7.37 ± 0.18 7.42 ± 0.38	8.41 ± 0.19 8.98 ± 0.32	8.10 ± 0.05 7.89 ± 0.58
**LVDD (mm**) week 0 week 8	8.97 ± 0.27 10.01 ± 0.18**^#^**	8.98 ± 0.16 9.30 ± 0.08	10.00 ± 0.24 10.95 ± 0.38	9.88 ± 0.10 10.06 ± 0.50
**FS (%**) week 0 week 8	22.00 ± 1.18 18.12 ± 2.37	16.60 ± 0.50 16.58 ± 1.90	15.37 ± 1.14 17.91 ± 0.71	18.00 ± 0.62 22.12 ± 2.56

Values are means ± SEM. Data analyzed by three-way ANOVA for repeated measurements (n=6-7 rats/group). LVEF: *time* F(1,23)=7.28, *P*=0.013; *group* F(1,23)= 0.84, *P*=0.370; *condition* F(1,23)=3.16, *P=*0.089; time × *group* F(1,23)=2.64, *P*=0.118; *time* × condition F(1,23)=0.002, *P=*0.960; group × condition F(1,23)=3.54, *P*=0.073; *time* × *group × condition* F(1,23)=0.113, *P*=0.740. LVSD: *time* F(1,23)=1.90, *P*=0.181; *group* F(1,23)= 13.97, *P=*0.001; *condition* F(1,23)=2.18, *P=*0.153; time *× group* F(1,23)=0.35, *P*=0.557; *time* × condition F(1,23)=2.96, *P=*0.099; group × condition F(1,23)=1.83, *P*=0.189; *time* × *group × condition* F(1,23)=0.001, *P*=0.982. LVDD: *time* F(1,23)=15.13, *P<*0.001; *group* F(1,23)=17.95, *P<*0.001; *condition* F(1,23)=4.01, *P=*0.057; time *× group* F(1,23)=0.14, *P*=0.712; *time* × condition F(1,23)=5.44, *P=*0.029; group × condition F(1,23)=0.13, *P*=0.723; *time* × *group × condition* F(1,23)=0.005, *P*=0.943. FS: *time* F(1,23)=0.37, *P=*0.547; *group* F(1,23)=0.001, *P=*0.983; *condition* F(1,23)=0.001, *P=*0.982; time × *group* F(1,23)=5.48, *P*=0.028; *time* × condition F(1,23)=1.45, *P=*0.241; group × condition F(1,23)=10.61, *P*=0.003; *time* × *group × condition* F(1,23)=0.26, *P*=0.618. Significances (*P*<0.05): ^#^ *vs*. week 0.

FS, fractional shortening. LVDD, left ventricle end diastolic diameter. LVEF, left ventricle ejection fraction. LVSD, left ventricle end systolic diameter.

HF rats treated with Veh and maintained sedentary (basal values of 102 ± 3 mmHg, 361 ± 18 b/min) exhibited higher vasomotor sympathetic activity and SAP variability, reduced cardiac parasympathetic control (HF-PI), and smaller PI variability when compared with the other experimental groups (Figure 2D, C, I and G, respectively). Both exercise training and Los reduced LF-SAP and increased HF-PI while augmenting the PI variability and decreasing the SAP variability exhibited by the sedentary Veh-treated HF rats. These effects did not change baseline HR and LF-PI values (Figure 2F and H) and were accompanied by significant increases in MAP and spontaneous baroreflex sensitivity (alpha HF) only in Veh-T groups (Figure 2B and E).

### Effects of exercise training and Los treatment on barrier permeability and transport mechanisms across the BBB in HF rats

**Figure 3:**
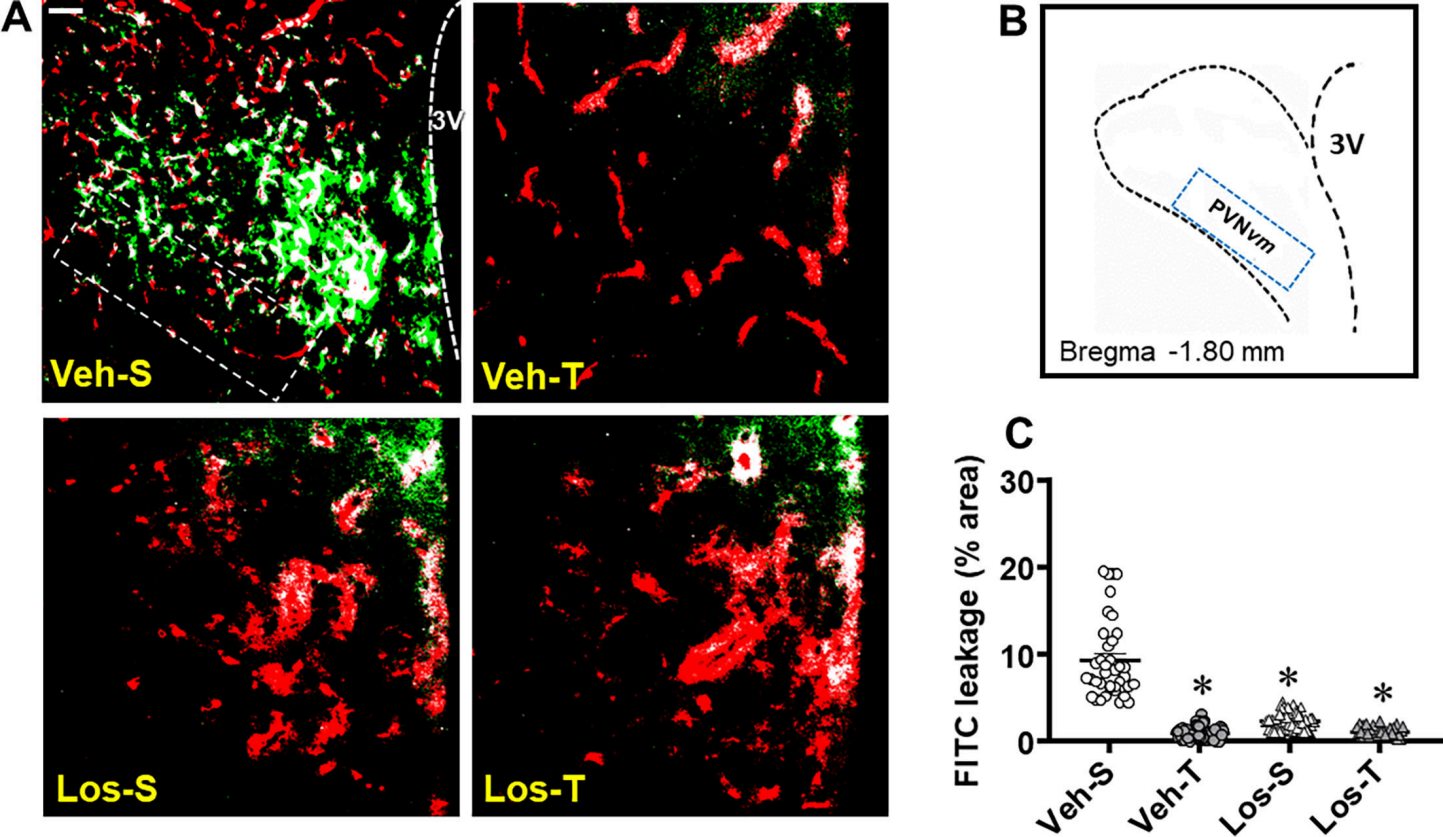
Blood–brain barrier permeability changes in heart failure rats treated with vehicle (Veh) or losartan (Los) and submitted to sedentary (S) or training (T) protocol. **(A)** Photomicrographs show the capillary network (Rhodamine-70kDa, red), the FITC-10kD leakage into the brain parenchyma (green), and the colocalization of both inside vessels (white) within the paraventricular nucleus of the hypothalamus (PVN). 3V, third ventricle, scale bar: 50 µm. (**B)** Illustrative figure with superimposed rectangle over the ventromedial nucleus (*vm*) indicates the ROI in which the measurements were made. (**C)** Effects of treatment and exercise on FITC-10kD leakage within the PVNvm. Values obtained in 10–12 slices/rat, three rats/group. Comparisons made by two-way factorial ANOVA: *group* F(1,122)=69.50, *P*<0.001; *condition* F(1,122)=116.10, *P*<0.001; *interaction* F(1,122)=70.64, *P*<0.001. Significance (*P*<0.05): ^*^vs. Veh-S.

BBB permeability was analyzed in anesthetized rats after the hemodynamic/autonomic recordings. Within the PVN*vm* (Figure 3A and B), the leakage of the low molecular weight dye (green dots) into the brain parenchyma exhibited by HF sedentary rats treated with Veh was almost absent in the other groups. Quantitative data confirmed a 10.2±0.8% area of FITC leakage in Veh-S group, which was abrogated by training, Los, and the combination of both (−88%, −77%, and −88%, respectively, Figure 3C).

**Figure 4:**
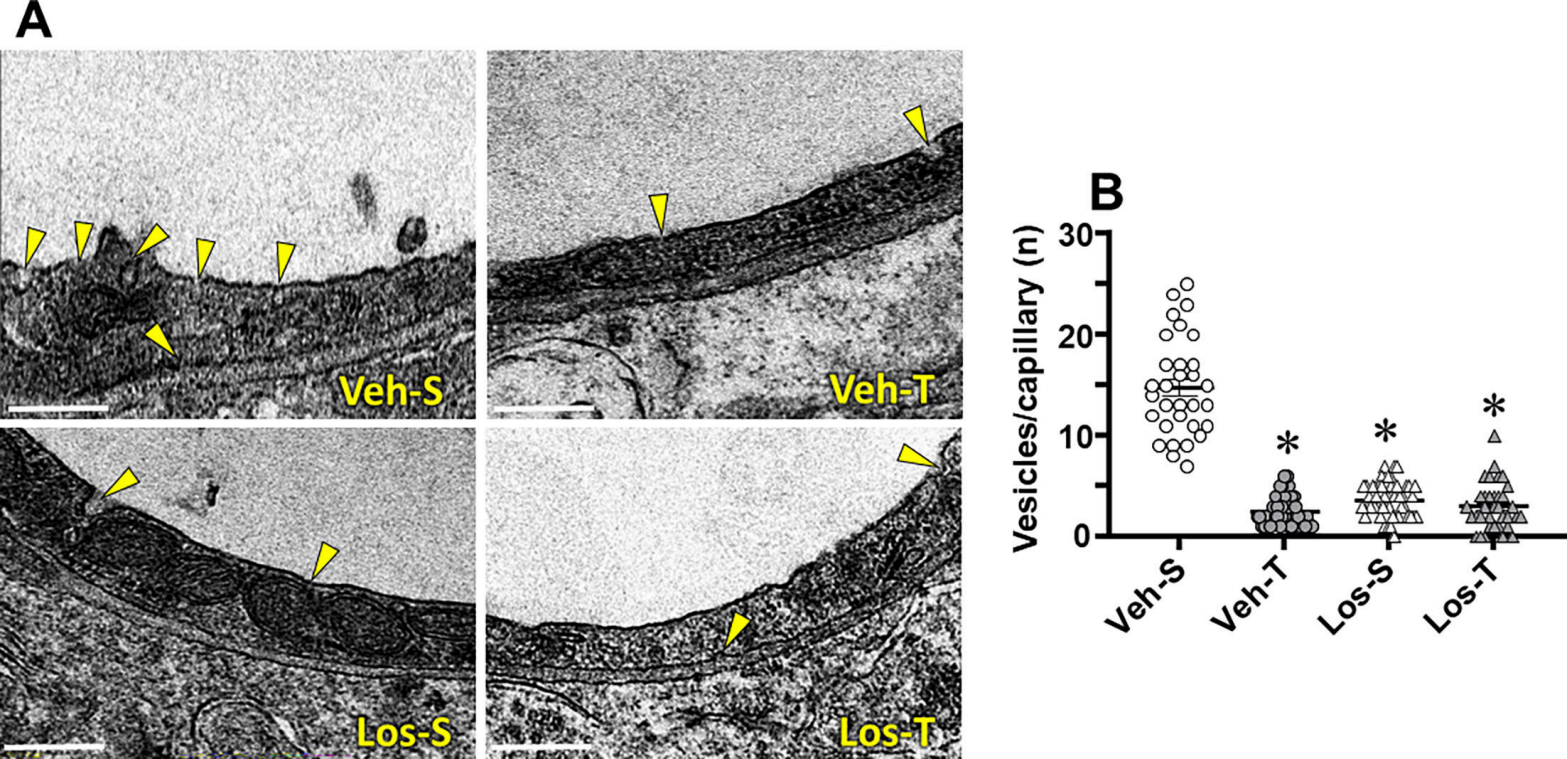
Transcellular vesicles/capillary in heart failure rats treated with vehicle (Veh) or losartan (Los) and submitted to sedentary (S) or training (T) protocol. (**A**) Electron microscopy photomicrographs depict the transcellular vesicles (yellow arrow heads) being formed in the luminal and abluminal borders of the capillary endothelial cell. Scale bar: 500 nm (**B)** Effects of treatment and exercise on vesicles’ number within PVN*vm* capillaries. values obtained in 12–15 capillaries/rat, three rats/group. Comparisons made by two-way factorial ANOVA. For *F* and *P* values, see Table 2. Significance (*P*<0.05): ^*^vs. Veh-S.

**Figure 5:**
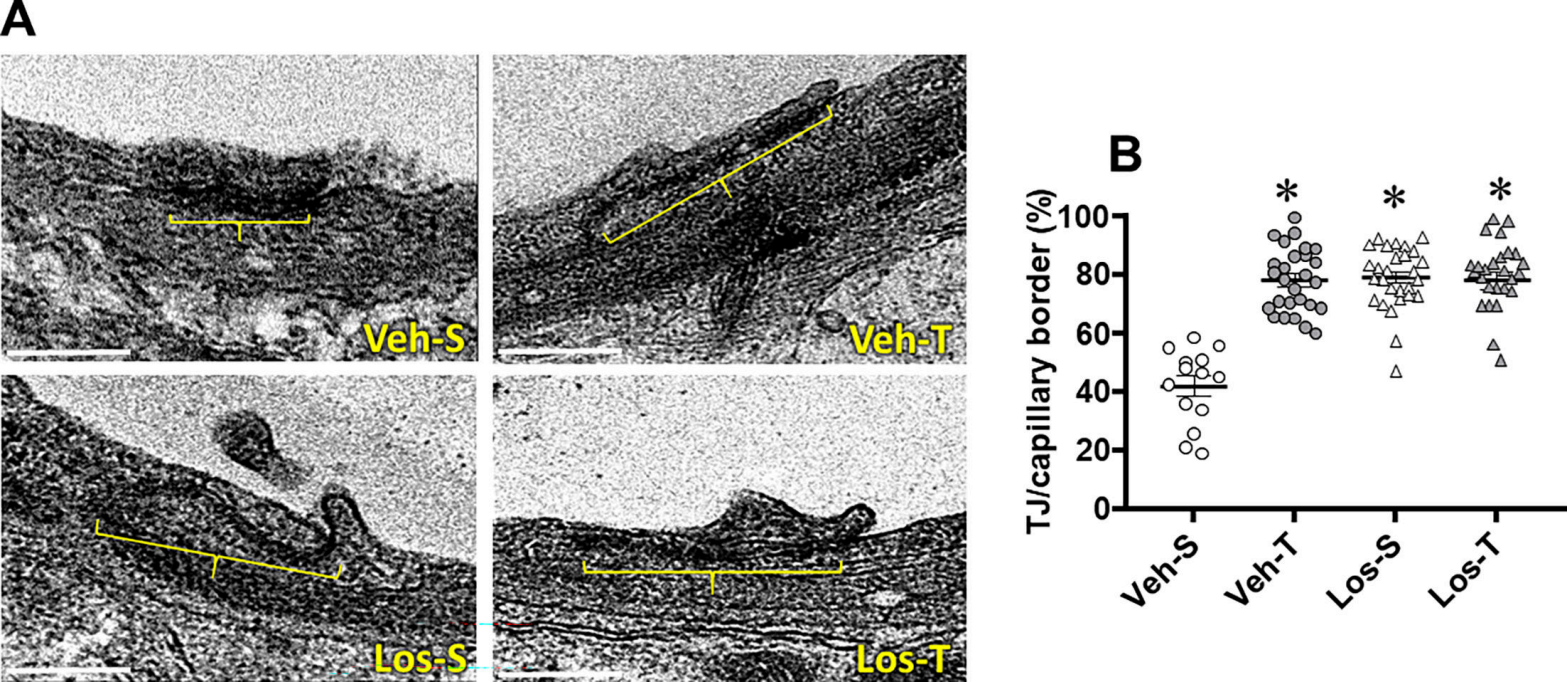
Tight junction (TJ) occupancy of capillary borders in heart failure rats treated with vehicle (Veh) or losartan (Los) and submitted to sedentary (S) or training (T) protocol. **(A)** Electron microscopy photomicrographs indicate the TJ extension (yellow bracket) in the border of neighboring endothelial cells. Scale bar: 500 nm. (**B)** Effects of Los and exercise on TJ extension/capillary border within PVN*vm* capillaries. values obtained in five to seven capillaries/rat, three rats/group. Comparisons made by two-way factorial ANOVA. For *F* and *P* values, see Table 2. Significance (*P*<0.05): ^*^vs. Veh-S. PVN, paraventricular hypothalamic nucleus.

The ultrastructure of BBB components within the PVN*vm* capillaries was evaluated and compared in the four groups of HF rats. The endothelial cell area of capillaries was not changed but the lumen diameter, reduced in Veh-S rats, was significantly augmented in the other 3 groups (average increases of 17% up to 29%, Table 2). Basement membrane thickness, larger in Veh-S rats, was reduced by exercise training, Los and the combination of both. Except for a small Los-induced increase in pericytes extension (group effect), pericytes’ coverage of the endothelial cell did not change (Table 2). Interestingly, as documented in Figure 4A, the counting of transcellular vesicles/capillary, higher in Veh-S group, was largely and similarly reduced by training, Los, and the combination of both (average reductions of 83%, 75%, and 80%, respectively, Table 2, Figure 4B), indicating a robust reduction in the absorptive transcytosis across the BBB. We also evaluated the effects of exercise and Los on the occurrence of TJs, responsible for the paracellular transport across the BBB: there was 0.61 ± 0.12 TJ/capillary in Veh-S rats, a value not altered in Los-S but slightly increased in trained groups (0.80 ± 0.10 and 0.80 ± 0.08 TJ/capillary, for Veh-T and Los-T respectively). By quantifying the ratio between TJ extension to capillary border extension between adjacent endothelial cells, we were able to analyze the effects of exercise and Los on TJ occupancy of the capillary border. There were slight fluctuations in both capillary border and TJ extension between groups (images in Figure 5A, values in Table 2), but the TJ/capillary border ratio was similarly augmented in Veh-T, Los-S, and Los-T groups (average increases of 87%, Table 2, Figure 5B), suggesting an important augmentation in their resistance to transport the hydrophilic substances across the paracellular pathway.

**Table 2:** Quantitative data of PVN capillaries’ ultrastructure in heart failure rats treated with vehicle (Veh) or losartan (Los) and submitted to aerobic training (T) or sedentary protocol (S).

PVN capillaries	Veh-S	Veh-T	Los-S	Los-T
Lumen diameter (μm)	5.2 ± 0.2	6.2 ± 0.2*	6.1 ± 0.2*	6.7 ± 0.3*
Endothelial cell area (μm^2^)	9.2 ± 1.0	9.6 ± 0.8	8.7 ± 0.9	9.1 ± 0.7
Basement membrane thickness (μm)	0.119 ± 0.005	0.080 ± 0.003*	0.076 ± 0.003*	0.082 ± 0.002*
Transcellular vesicles/capillary (n)	14.8 ± 0.9	2.5 ± 0.2*	3.7 ± 0.2*	3.0 ± 0.4*
Capillary border extension (μm)	1.83 ± 0.38	1.43 ± 0.12	1.09 ± 0.14*	0.96 ± 0.06*
Tight Junction extension (μm)	0.77 ± 0.17	1.14 ± 0.11	0.87 ± 0.11	0.74 ± 0.05**^†^**
Tight Junction/capillary border (%)	42 ± 3	78 ± 3*	79 ± 2*	78 ± 3*
Pericytes’ coverage (%)	30 ± 2	28 ± 1	35 ± 2	36 ± 2**^†^**

Values are mean ± SEM and were obtained from 31 to 47 capillaries/group analyzed in three rats/group. Data analyzed by two-way factorial ANOVA. Lumen diameter: *group* F(1,140)=6.89, *P*=0.001; *condition* F(1,140)=10.79, *P*<0.001; *interaction* F(1,140)=0.51, *P*=0.477. Endothelial cell area: *group* F(1,140)=0.26, *P=*0.609; *condition* F(1,140)=0.19, *P=*0.661; *interaction* F(1,140)=0.00, *P=*0.996. Basement membrane thickness: *group* F(1,144)=44.24, *P*<0.001; *condition* F(1,144)=27.75, *P*<0.001; *interaction* F(1,144)=54.83, *P*<0.001. Transcellular vesicles/capillary: *group* F(1,150)=142.00, *P*<0.001; *condition* F(1,150)=209.90, *P*<0.001; *interaction* F(1,150)=169.30, *P*<0.001. Capillary border extension: *group* F(1,92)=13.98, *P*<0.001; *condition* F(1,92)=2.55, *P*=0.114; *interaction* F(1,92)=0.69, *P*=0.401. Tight junction extension: *group* F(1,92)=1.73, *P=*0.192; *condition* F(1,92)=1.12, *P=*0.292; *interaction* F(1,92)=5.02, *P=*0.027. Tight junction/capillary border: *group* F(1,92)=47.38, *P*<0.001; *condition* F(1,92)=43.04, *P*<0.001; *interaction* F(1,92)=47.96, *P*<0.001. Pericytes` coverage: *group* F(1,139)=14.17, *P=*0.001; *condition* F(1,139)=0.08, *P=*0.780; *interaction* F(1,139)=0.87, *P*=0.350. Significances (*P* <0.05) are: * *vs.* Veh-S; ^†^ *vs.* Veh-T.

### Effects of exercise training and Los treatment on brain Ang II availability, microglia expression, and their colocalization within the PVN of HF rats

**Figure 6:**
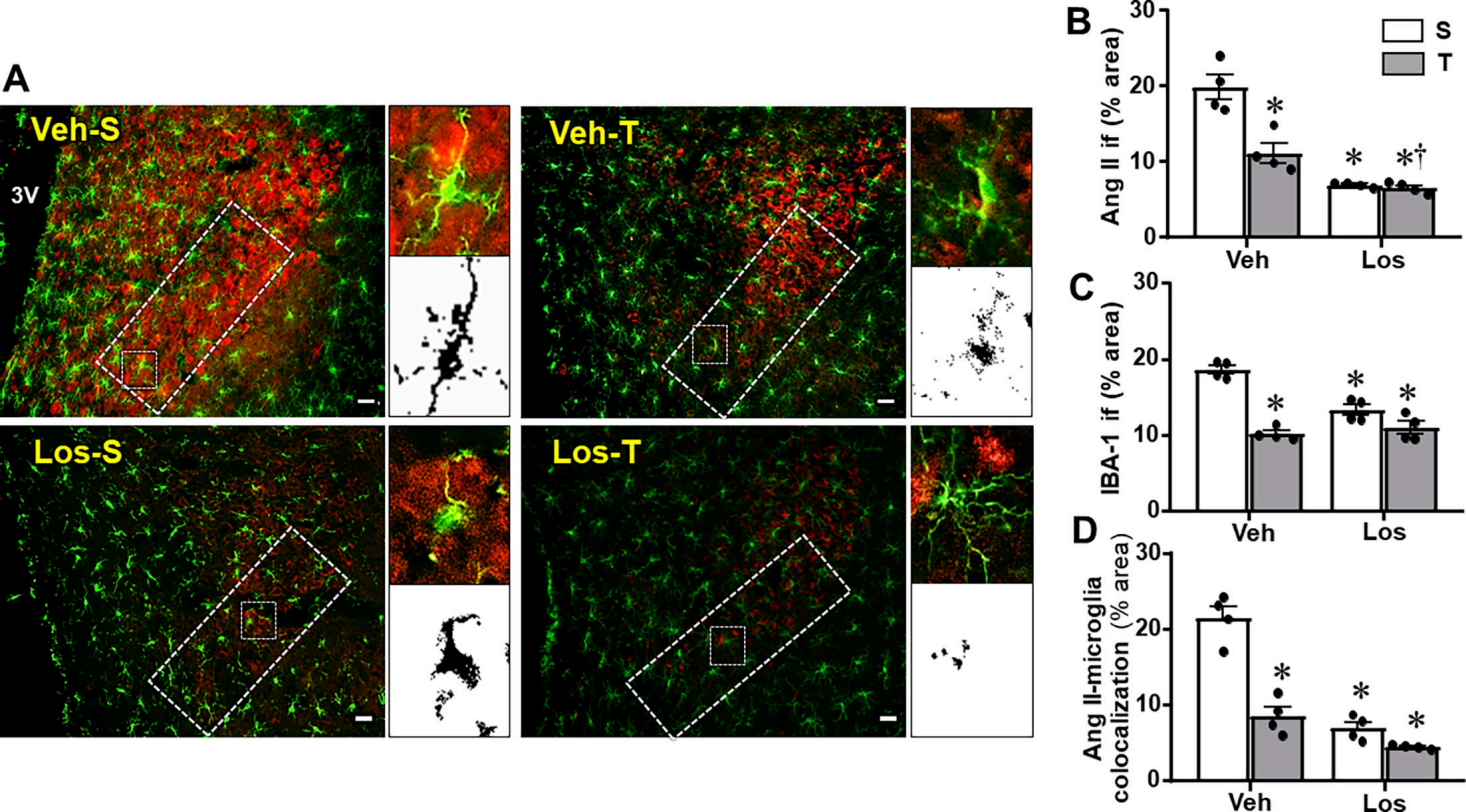
Angiotensin II (Ang II) and microglia (IBA-1) expression and the colocalization of both in heart failure rats treated with vehicle (Veh) or losartan (Los) and submitted to sedentary (S) or training (T) protocol. **(A)** Representative PVN images illustrating Ang II (red) and IBA-1 immunoreactivity (green) within the ventromedial nucleus (superimposed rectangle) in which measurements were made. The small square indicates the microglial cell whose magnified images (insets at right) depict Ang II-microglia colocalization (yellow) in soma and processes as analyzed in merged pictures (upper corner) or quantified by means of superimposed binary images (lower corner). Scale bar: 50 μm. 3V, third cerebral ventricle. Bar graphs at the right indicate the quantitative analysis of Ang II (**B**) and IBA-1 (**C**) immunoreactivity and the colocalization of both (**D**). Comparisons made by two-way factorial ANOVA. Values are means of six to eight slices/rat/four rats/group. ang II: *group* F(1,12)=67.98, *P*<0.001; *condition* F(1,12)=18.41, *P=*0.001; *interaction* F(1,12)=15.38, *P*=0.002. IBA-1: *group* F(1,12)=13.93, *P=*0.003; *condition* F(1,12)=79.23, *P<*0.001; *interaction* F(1,12)=25.47, *P*<0.001. Colocalization: *group* F(1,12)=74.00, *P*<0.001; *condition* F(1,12)=50.67, *P<*0.001; *interaction* F(1,12)=23.48, *P*<0.001. Significances (*P*<0.05): ^*^vs. Veh-S; **^†^** *vs*. Veh-T. PVN, paraventricular hypothalamic nucleus.

**Figure 7:**
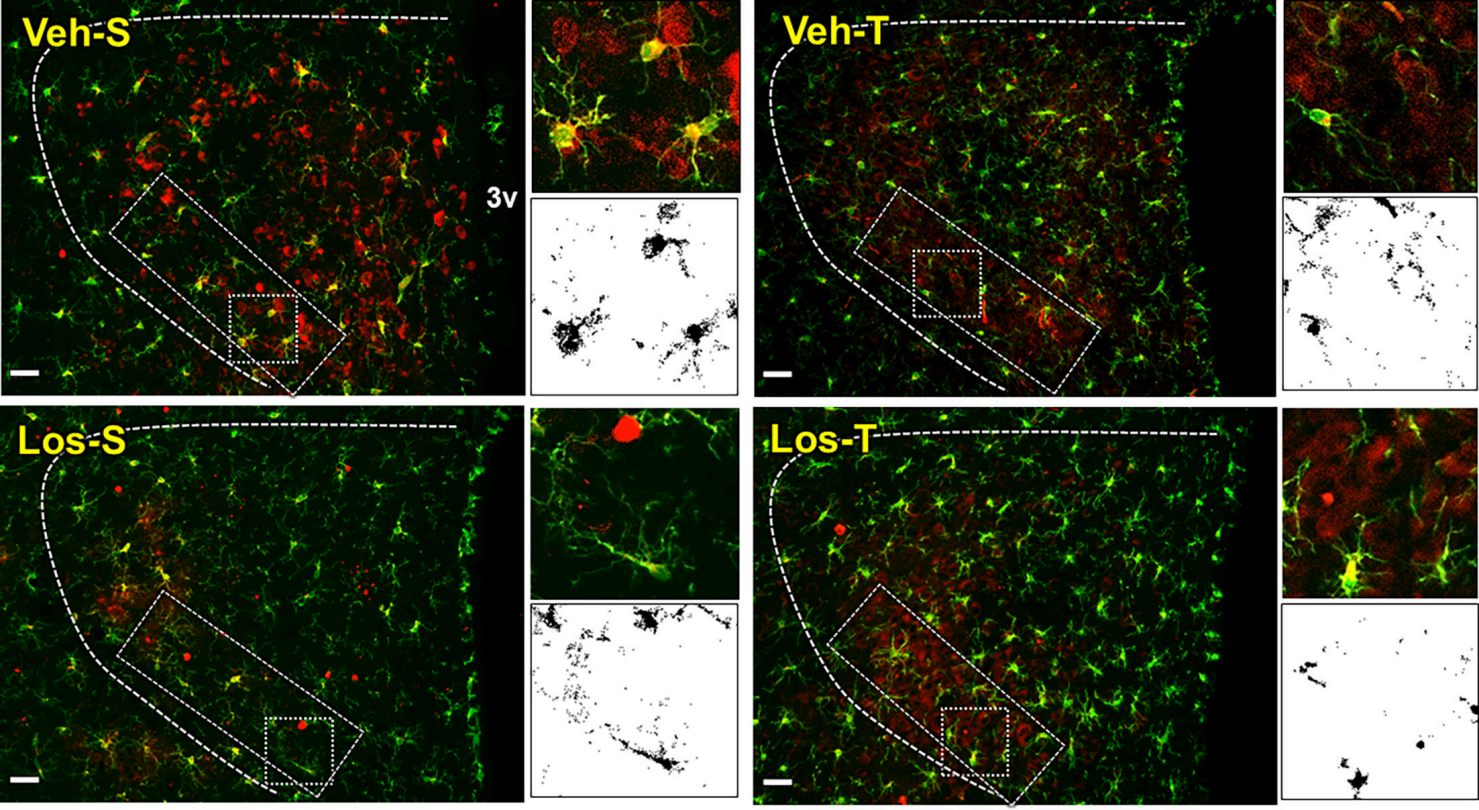
**Angiotensin type 1 receptor (AT**_**1**_ **R) expression in microglial cells of heart failure rats treated with vehicle (Veh) or losartan (Los) and submitted to sedentary (S) or training (T) protocol.** Representative PVN images illustrating AT_1_R (red) and IBA-1 immunoreactivity (green) within the within the PVN*vm* (superimposed rectangle). The small square indicates the microglial cell whose magnified images (insets at right) depict Ang II-microglia colocalization (yellow) in soma and processes in merged pictures (right upper corner) and the superimposed binary images (right lower corner). Scale bar: 50 μm. 3V, third cerebral ventricle; PVN, paraventricular hypothalamic nucleus.

As shown in Figure 6A, there was high Ang II expression within the PVN of sedentary Veh-treated HF rats that exhibited increased transcytosis, reduced TJs tightness, and augmented BBB permeability (Figures 4B, 5B and 3C, respectively). Quantitative data confirmed the increased Ang II availability within the PVN*vm* of Veh-S rats and the robust reductions in its expression in Veh-T, Los-S, and Los-T groups (decreases of 44%, 65%, and 67%, respectively (Figure 6B) in which the altered transcytosis, TJs’ weakness, and elevated BBB permeability were corrected. IBA-1 immunofluorescence was also elevated in Veh-S rats with significant reductions in the other three groups (average decreases of 46%, 29%, and 41%, respectively, Figure 6C). Right insets in Figure 6A depict augmented images of microglial cells representative of the 4 experimental groups illustrating the colocalization of Ang II within the soma and processes (merged signals, right upper corner) and the respective binary images used to quantify their colocalization (right lower corner). As expected, the association of Ang II with microglial cells was higher in sedentary HF rats treated with Veh and largely reduced by exercise, Los, and the combination of both (decreases of 60%, 68%, and 79% in Veh-T, Los-S and Los-T groups, respectively, Figure 6D). To corroborate the microglia activation by Ang II, double-labelled slices from one rat/group were processed for AT_1_R and IBA-1 immunofluorescence and their binary images analyzed by the command ‘colocalization’ of the ImageJ (Figure 7). AT_1_R expression observed within several microglial cells in Veh-S rat was largely reduced in trained rats treated with Veh or Los. Right insets in Figure 7 compare, for the four experimental groups, magnified images of microglial cells showing AT_1_R colocalized mainly in the soma (merged yellow images at the upper inset, black dots of the binary images at the lower inset) showing that besides the smaller number of colocalized cells, exercise and Los also reduced AT_1_R expression in each microglial cell.

### Effects of exercise training and Los treatment on microglia morphological states within the PVN of HF rats

**Figure 8:**
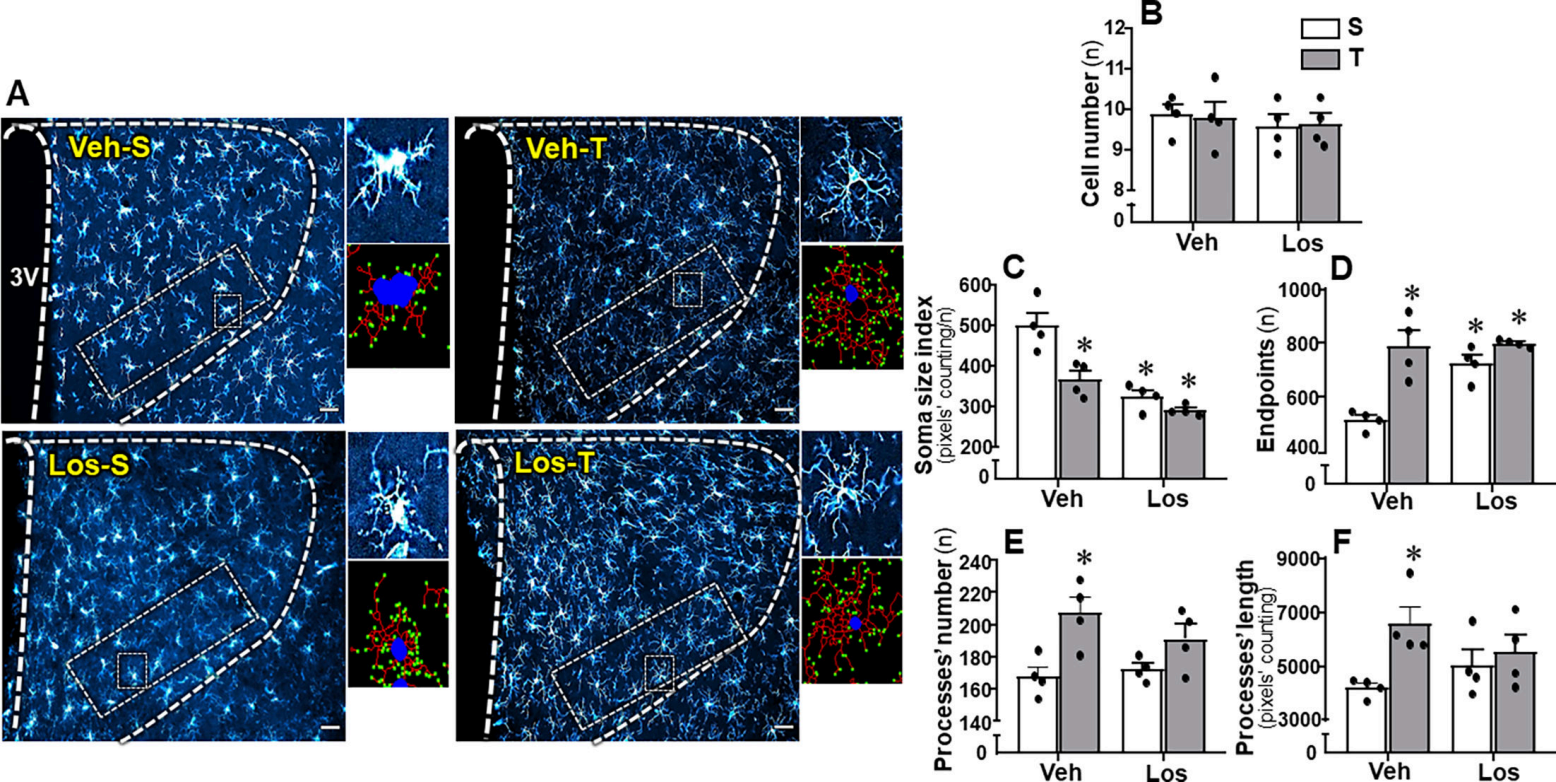
Morphological changes of microglia in heart failure rats treated with vehicle (Veh) or losartan (Los) and submitted to sedentary (S) or training (T) protocol. **(A)** PVN images depicting IBA-1 immunoreactivity (blue) in rats representative of their experimental groups. The superimposed rectangle over the PVN*vm* indicates the ROI in which measurements were made; the small square indicates the microglial cell whose magnified image (insets at right) shows its morphology (upper corner) or the analysis of soma size (dark blue), processes number/length (red), and endpoints (green) quantified by means of NeurphologyJ plugin to ImageJ (lower corner). Scale bar: 50 μm. 3V, third cerebral ventricle. Bar graphs at the right indicate the quantitative analysis of cell number (**B**), soma size index (**C**), endpoints (**D**), processes number (**E**), and length (**F**). Comparisons made by two-way factorial ANOVA. Values are means of six to eight slices/rat/four rats/group. Cell number: *group* F(1,12)=0.58, *P=*0.461; *condition* F(1,12)=0.02, *P=*0.881; *interaction* F(1,12)=0.02, *P*=0.881. Soma size index: *group* F(1,12)=23.60, *P<*0.001; *condition* F(1,12)=10.45, *P=*0.007; *interaction* F(1,12)=3.67, *P*=0.080. Endpoints: *group* F(1,12)=9.78, *P=*0.009; *condition* F(1,12)=24.32, *P<*0.001; *interaction* F(1,12)=7.78, *P*=0.016. Processes number: *group* F(1,12)=0.38, *P=*0.551; *condition* F(1,12)=11.79, *P=*0.005; *interaction* F(1,12)=1.82, *P*=0.202. Processes length: *group* F(1,12)=0.04, *P=*0.841; *condition* F(1,12)=6.69, *P=*0.024; *interaction* F(1,12)=2.82, *P*=0.119. Significances (*P*<0.05): ^*^vs. Veh-S. PVN, paraventricular hypothalamic nucleus.

Changes in IBA-1 density (high in Veh-S and lower in the other groups, Figure 6C) were accompanied by alterations in the morphological phenotype of microglial cells (see the amplified images on right insets of Figure 8A), which is an index of their surveillant or activated state. Microglial cells of Veh-S HF rats exhibited enlarged soma, short process arbors and few terminal ramifications, a pattern characteristic of activated microglia. A completely different pattern was observed in the other three experimental groups that exhibited smaller soma size, numerous and ramified processes that characterize the homeostatic microglia (Figure 8A). Quantitative analysis confirmed a significant reduction in the soma size in Veh-T, Los-S, and Los-T groups (−27%, −32%, and −42%, respectively, when compared with Veh-S, Figure 8C). In addition, both exercise training and Los treatment increased microglia endpoints ( + 53%, + 41% and + 56% in Veh-T, Los-S and Los-T rats, respectively, Figure 8D). Detailed analysis performed by the NeurphologyJ also showed that exercise training increased the processes number and length of microglial cells in Veh-T group ( + 23% and + 56%, respectively, Figure 8E and F). Interestingly, the small changes of processes’ number and length exhibited by microglial cells in Los-treated rats did not attain significance. Although the number of microglial cells within the PVN*vm* was not altered by experimental procedures (Figure 8B), the morphologic phenotype changes observed indicated a transition from disease-associated in Veh-S to homeostatic-surveilling conditions in the other experimental groups.

### HF-, exercise-, and Los-induced changes on BBB permeability, microglia activation, and autonomic control correlate with brain Ang II availability

**Figure 9:**
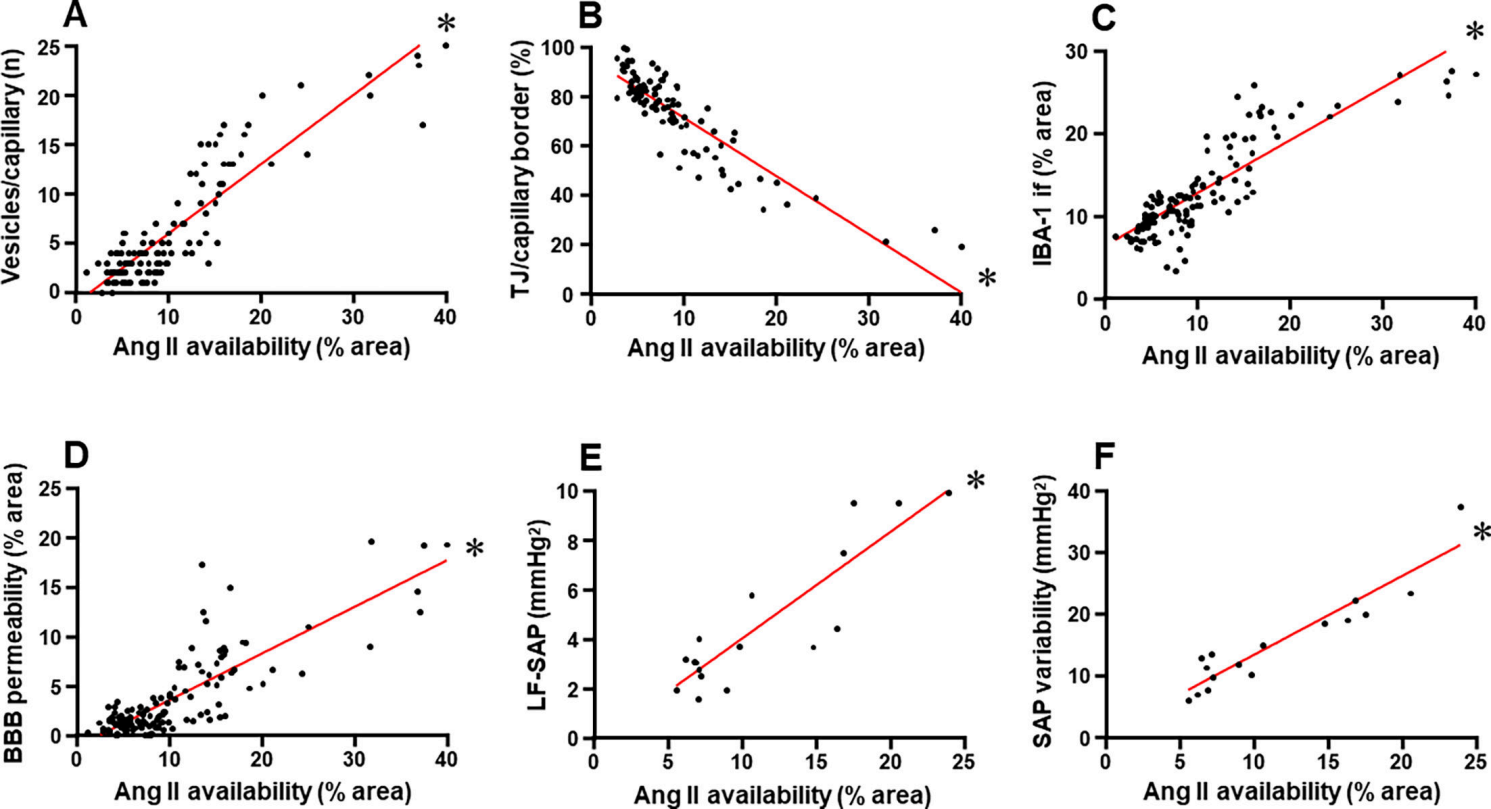
Correlations between BBB permeability, transport mechanisms across the barrier, microglia activity, and autonomic control with angiotensin II (Ang II) availability within the PVNvm in heart failure rats treated with vehicle or losartan and submitted to sedentary or training protocol. Plots in panels A–D were made with values obtained in a large number of capillaries, Ang II, and IBA-1 double-labeled slices, whereas panels E and F were made with autonomic measurements in each rat and its respective mean Ang II immunoreactivity. Linear regression equations, correlation coefficients, and *P* values are Vesicles/capillary × Ang II Y = 0.70 × –1.12, *r* = 0.895, *P*<0,001; TJ/capillary border × Ang II Y = –2.36 × + 94.94, *r* = 0.881, *P*<0,001; IBA-1 if × Ang II Y = 0.64 × + 6.29, *r* = 0.855, *P*<0,001; FITC leakage × Ang II Y = 0.47 × –1.18, *R* = 0.824, *P*<0,001; LF-SAP × Ang II Y = 0.43 × –0.34, *R* = 0.889, *P*<0,001; SAP variability × Ang II Y = 1.27 × + 0.53, *r* = 0.935, *P*<0,001. * denotes a significant correlation. BBB, blood–brain barrier; SAP, systolic arterial pressure; TJ, tight junction.

The present set of data showed that Ang II availability within the PVN*vm* was negatively correlated with TJ tightness and positively correlated with both absorptive transcytosis and microglia density (Figure 9B, A and C, respectively). Dysfunctional transport mechanisms and microglia activation determined not only a marked augmentation in BBB permeability when PVN Ang II content increases but also increased the sympathetic activity, autonomic imbalance, and pressure variability, as ratified by the strong positive correlations (Figure 9D, E and F).

## Discussion

Our data in sedentary HF rats treated with Veh corroborate previous findings on BBB dysfunction, microglia activation, and autonomic imbalance [9]. Additionally, new original observations were made (i) the elevated BBB permeability within the PVN of HF rats is equally corrected by exercise training, Los, or a combination of both; (ii) the normalization of BBB function in Veh-T, Los-S, and Los-T groups is attained by marked reductions in absorptive transcytosis associated with increased TJs’ tightness; (iii) changes in microglia density and their transition from disease-associated in Veh-S rats to homeostatic-surveillant condition are similar in trained and Los-treated HF rats; (iv) the high expression of Ang II in HF rats treated with Veh is similarly abrogated by exercise training, Los or a combination of both; (v) PVN Ang II expression exhibits strong correlations with BBB permeability, microglia activation, and autonomic control of the circulation indicating that local Ang II availability is a key stimulus to drive not only the deleterious effects of HF (high levels) but also the beneficial remodeling induced by exercise training (low Ang II levels).

It is well known that HF is characterized by cardiac dysfunction, abnormal parasympathetic responsiveness, sympathoexcitation, autonomic imbalance, and a compensatory activation of plasma and tissue RAS [1–5,16]. We [9] recently demonstrated that HF depresses BBB function within autonomic nuclei, alters the transport mechanisms across the capillary, and augments the barrier’s permeability. However, the mechanism(s) modulating transcellular and paracellular transport across the BBB in HF animals remained to be determined. In chronic hypertension, Biancardi et al. [13] demonstrated the participation of Ang II in BBB lesion, which was alleviated by Los treatment. In trained SHR simultaneously infused with intracerebroventricular Ang II, we also showed a complete blockade of exercise-induced correction of BBB leakage [14] and that plasma Ang II, by gaining access into the brain parenchyma via the absorptive transcytosis, contributed to BBB dysfunction [12,20]. Accordingly, the present set of data confirmed in Veh-treated sedentary HF rats the high Ang II expression into the PVN, which was accompanied by increased vesicles trafficking, reduced TJs tightness, increased BBB permeability, activated microglial cells, and high sympathetic activity. Over-activation of RAS vasoconstrictor axis is well stablished in the pathogenesis of HF [1,10,11]. Increased Ang II expression in chronic HF activates Ang II-AT1 receptor cascade, the upstream modulator of oxidative stress, inflammation, and microglia activation. Previous studies have already shown that either, oxidative stress, pro-inflammatory cytokines secretion and microglia activation result in BBB disruption [6,7,23,24]. Indeed, the Ang II not only activates microglial cells but also the Ang II-AT_1_R signaling cascade leading to a series of downstream effects as the activation of NAD(P)H oxidase, oxidative stress, and pro-inflammatory genes expression, as well as the up-regulation of NF-κB, which activate immune-mediated mechanisms, the accumulation of reactive oxygen species and the synthesis/release of pro-inflammatory cytokines [25,26]. Previous studies in chronic HF have already shown that blockers of the vasoconstrictor axis of the RAS as well as exercise training reduce central Ang II signaling, oxidative stress, and inflammation, thus reducing the sympathoexcitation and correcting the autonomic imbalance [4,5,10,11,25–27]. It is likely that these downstream effects of Ang II–AT_1_R pathway modulated the responses now observed in Veh-, Los-, and exercise-treated HF rats.

An important observation of our previous HF study was the high efficacy of exercise training in correcting BBB permeability and restoring both the normal barrier function and the autonomic control of the circulation even in the persistence of cardiac dysfunction [9]. Although experimental evidence confirmed that converting enzyme inhibitors, AT_1_R blockers, and exercise training down-regulated RAS and normalized Ang II-induced oxidative and pro-inflammatory effects in HF animals [1,10,11,25–27], there was no information on its effects on transport mechanisms across the BBB. The present set of data expanded our knowledge showing that both exercise and Los caused similar reductions in PVN Ang II availability and absorptive transcytosis, as well as similar increases in TJs’ tightness, thus determining robust and equal reductions in FITC leakage into the brain parenchyma. The association of exercise and Los treatment had no additional effect in HF rats, suggesting that the blockade of Ang II-AT_1_R cascade is the main mechanism underlying the correction of BBB permeability. We also observed that both stimuli not only blocked the high expression of Ang II within the PVN*vm* of HF rats but reduced the local expression of AT_1_R. Attenuation of AT_1_R expression following Los treatment was already observed into the PVN of HF rats [25]. Indeed, Haack et al. [27] documented that exercise training decreased the phosphorylation of G-protein-coupled receptor kinase-5, reduced both AT_1_R internalization and NF-κB expression, determining AT_1_R down-regulation within the PVN of HF rats. It is likely that these downstream effects of Ang II–AT_1_R pathway modulated the responses now observed following exercise training and Los treatment.

In addition to the above-mentioned effects, sedentary HF rats treated with Veh exhibited high density of activated microglia within the PVN*vm,* as indicated by their morphological phenotype. Recent studies also reported structural remodeling of activated microglia (small cell volume, reduced branches, shorter processes, decreased terminal ramifications) associated with increased pro-inflammatory cytokines expression (TNFα, IL-1β, IL-6) within the PVN of HF rats, as indicated by their altered morphology [6,7]. It is well known that activated microglia is an important factor to increase sympathoexcitation [28–30]. Importantly, the present set of data showed that exercise training, Los, and the combination of both caused significant and similar reductions on microglial cells immunofluorescence and determined their remodeling from disease-associate to homeostatic-surveilling condition. These effects were mediated by local Ang II expression, as confirmed by changes in the colocalization of both Ang II and AT_1_R with microglial cells, which were higher in Veh-S, but lower in the other three experimental groups. AT_1_R up-regulation in activated microglial cells, its reversal after Los treatment, and improvement of neuronal function have already shown in HF rats [7]. In accordance, our data confirmed the effects of Los showing in addition that exercise by itself not only decreased Ang II content and AT_1_R expression and corrected the increased transcytosis and TJs’ weakness but also caused the transition from activated microglial cells in Veh-S rats (enlarged soma, fewer endpoints) to reduced soma size and increased number of terminal ramifications in the other groups, a pattern characteristic of homeostatic-surveillant cells. Moreover, microglial cells exhibited a more complete remodeling of their morphologic phenotype after the exercise training showing increased processes’ number and length, effects that were absent in Los-treated HF rats. It should be noted that in contrast to inflammatory and neurodegenerative diseases that involve intense microglial proliferation, HF, a low-grade inflammatory disease, did not change the number of microglial cells. Similar observation was made in SHR, another low-grade inflammatory disease [12]. Therefore, exercise- and Los-induced establishment of homeostatic/surveillant phenotype in PVN microglial cells led to subsequent reduction in local oxidative stress and inflammatory profile [6,30] and improved the autonomic control of the circulation, as documented by the decreased vasomotor sympathetic activity, increased parasympathetic control of the heart, augmented PI variability, and reduced pressure variability. Importantly, all the observed effects on BBB permeability changes, microglia density/activity, as well as the autonomic control exhibited strong correlations with Ang II availability into the PVN, the key regulator of these responses in HF.

A caveat of this study is that experiments were only made in male rats. However, men and women, as well as male and female rats, are prone to develop HF. A differential balance exists in pressor and depressor arms of the RAS in males and females, but both sexes respond to RAS inhibition [31]. Moreover, studies on BBB function made in male and female pups and embryonic brains did not find sex-based differences [32,33]. Although HF-, Los-, and exercise-induced effects remain to be confirmed, we believe that similar BBB dysfunction and exercise-induced correction also characterizes the HF in female rats.

## Conclusions

**Figure 10:**
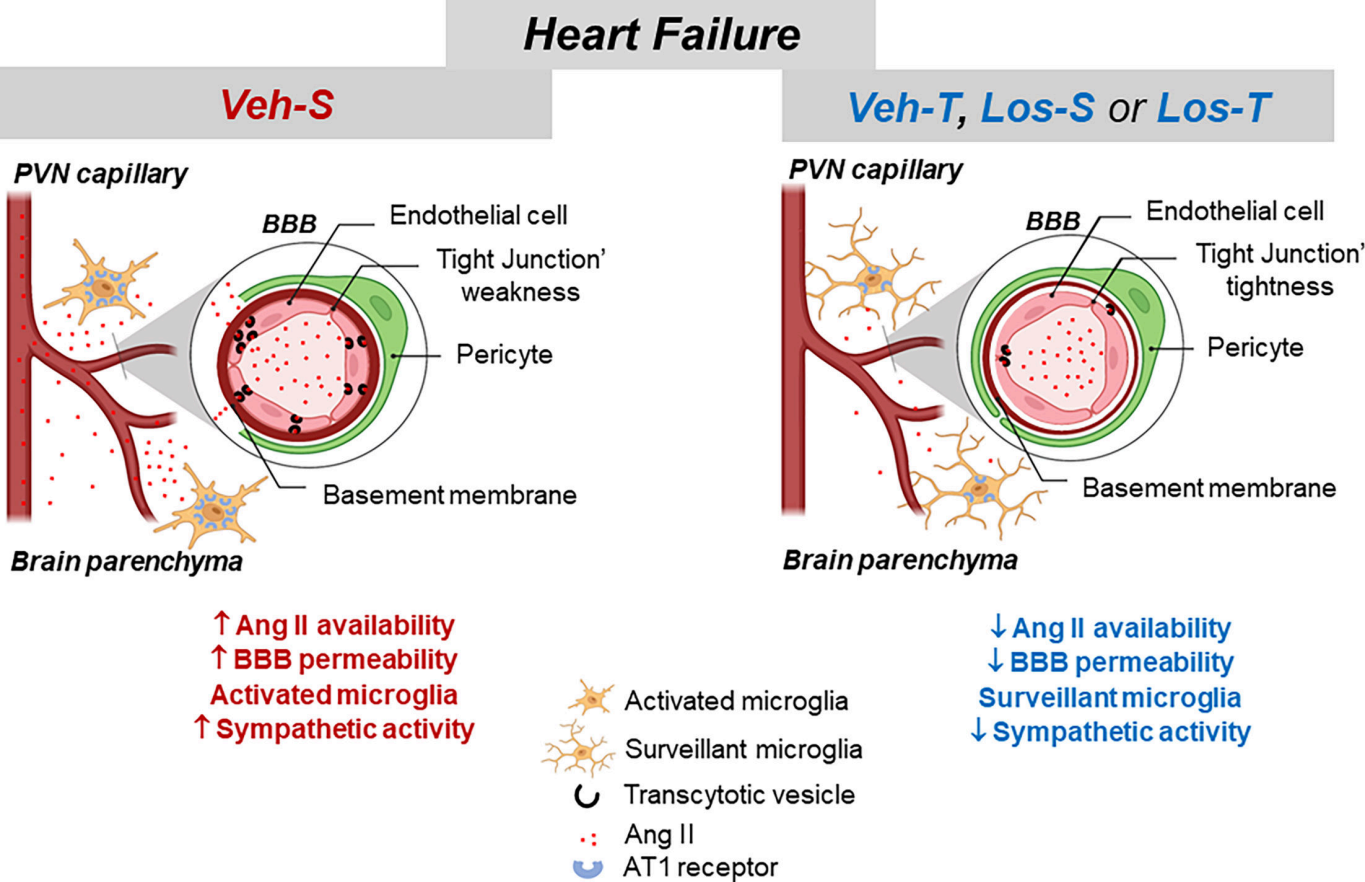
Schematic drawing depicting main effects of angiotensin II availability on blood–brain barrier (BBB), microglia morphology/activity, and sympathetic activity in heart failure (HF) rats treated with vehicle (Veh) or losartan (Los) and submitted to sedentary (S) or training (T) protocol.

As depicted in Figure 10, high tissue Ang II in sedentary HF rats treated with Veh is accompanied by BBB leakage and activates a vicious cycle by which the continuous entrance of plasma Ang II into the brain parenchyma augments its local availability and potentiates BBB dysfunction. Resident microglial cells are also activated by increased brain Ang II levels contributing to augment the sympathoexcitation that characterizes HF. In contrast, exercise training and Los, by abolishing the entrance of plasma Ang II into the brain parenchyma and blocking its effects, correct both the elevated absorptive transcytosis and TJs’ weakness, thus reducing BBB permeability. The maintenance of low brain Ang II levels also drives the remodeling of microglial cells towards homeostatic-surveillant state and the robust reduction in vasomotor sympathetic activity even in the persistence of cardiac dysfunction. Together, our data indicated that PVN Ang II availability induced by HF, exercise, and Los is the key regulator of BBB function, microglia reactivity, and autonomic control of the circulation.

**Clinical Perspectives**

- Reduced ventricular function, up-regulation of the renin–angiotensin system and sympathoexcitation are hallmarks of heart failure (HF). Blood–brain barrier (BBB) lesion within autonomic nuclei, which allows the entrance of plasma constituents into the brain parenchyma and contributes to autonomic dysfunction, is corrected by exercise training. Our knowledge on the mechanisms conditioning these effects is elusive.
- Increased absorptive transcytosis, tight junctions’ weakness, and the leaky BBB observed within paraventricular hypothalamic nucleus (PVN) capillaries of HF rats occurred simultaneously with high local angiotensin II (Ang II) expression and microglia activation. Exercise training, as well as losartan (Los), reduces PVN Ang II availability, remodels microglial cells from disease-associated phenotype to homeostatic-surveilling condition, corrects BBB deficits, protects neuronal processing, and improves autonomic control even in the persistence of cardiac dysfunction. The association of exercise and Los causes no additional effects.
- Data indicate that changes in Ang II availability within autonomic nuclei induced by HF, exercise, and Los is the key regulator of BBB function, microglia reactivity, and autonomic control of the circulation. Data also emphasize the equal effectiveness of exercise and AT_1_R blockade to correct HF-induced deficits and improve cardiovascular fitness in HF patients.

## Data Availability

The data that support the findings of the present study are available from the corresponding author upon reasonable request.

## Open Access

This article has been published open access under our Subscribe to Open programme, made possible through the support of our subscribing institutions, learn more here: https://portlandpress.com/pages/open_access_options_and_prices#conditional

## Abbreviations

AT_1_R: angiotensin II type 1 receptor
Ang II: angiotensin II
BBB: blood–brain barrier
HF: heart failure
HR: heart rate
Los: losartan
MAP: mean arterial pressure
PI: pulse interval
PVN: paraventricular hypothalamic nucleus
PVNvm: ventromedial subnucleus of the PVN
RAS: renin–angiotensin system
S: sedentary
SAP: systolic arterial pressure
T: trained
TJ: tight junction
Veh: vehicle
